# Environmental Performance and Economic Growth in Middle East and North Africa Countries

**DOI:** 10.5696/2156-9614-9.24.191208

**Published:** 2019-11-27

**Authors:** Hichem Dkhili

**Affiliations:** 1 College of Business Administration, Northern Border University, Arar, Saudi Arabia; 2 Faculty of Law and Management, University of Jendouba, Jendouba, Tunisia

**Keywords:** environmental performance, economic growth, MENA countries, PSTR model

## Abstract

**Background.:**

Studies on environmental performance/quality and economic growth show inconclusive results.

**Objective.:**

The aim of the present study is to assess the non-linear relationship between environmental performance and economic growth in the Middle East and North Africa (MENA) region from 2002–2018.

**Methods.:**

A sample of fourteen (14) MENA countries was used in the present analysis. However, due to important differences between countries in this region, the whole sample was divided into two sub-samples; nine Middle Eastern countries (MEAS) and five North African countries (NAF). We performed the panel smooth transition regression model as an econometric approach.

**Discussion.:**

Empirical results indicate a threshold effect in the environmental performance and economic growth relationship. The threshold value differs from one group of countries to another. More specifically, we found that the impact of environmental performance and economic growth is positive and significant only if a certain threshold level has been attained. Until then, the effect remains negative.

**Conclusions.:**

The findings of the present study are of great importance for policymakers since they determine the optimal level of environmental performance required to act positively on the level of economic growth. MENA countries should seek to improve their environmental performance index in order to grow output.

**Competing Interests.:**

The authors declare no competing financial interests.

## Introduction

Environmental performance is a very important concern around the world. Due to recent climate change, this topic has attracted the attention of policymakers and researchers.^1^ Several studies have argued that the environment could be considered to be an input of production.^2^ The environment contains natural and geographical infrastructures that can influence economic growth and economic development.^3^ The work of Grossman and Krueger demonstrated that in the early stages of economic growth, pollution increases, but decreases under high-income levels.^4^ This trend supports an inverted U-shaped relationship in the real income and pollution relationship.

Socioeconomic status appears to have positive and negative associations with health.^5^ Economic growth contributes to a healthy population by providing the means to meet essential needs such as food, clean water and shelter, as well access to basic health care services.^6^ Health inequities are well documented, but their economic dimensions have received less attention.^7^

### Literature review

Studies on environmental performance/quality and economic growth show inconclusive results. Many studies support a positive association between environmental performance and economic growth.^8–13^ On the contrary, a negative association between environmental quality and growth or openness has been also supported by several studies.^9^,^12^,^14^,^15^ This topic has been studied in two channels: from environmental performance to economic growth and/or from growth and trade openness to environmental quality. Several studies have been conducted on the effect of growth and openness on environmental performance. Most of these studies concluded that trade and growth harm environmental quality.^16^,^17^ In addition to these positive or negative associations, some studies found no evidence between environmental performance and economic growth.^4^,^18^

Studies that support the positive relationship between environmental performance/quality and economic growth include that of Maji, who explored the linkage between economic growth, trade liberalization and environmental quality in Nigeria from 1981–2011.^11^ The main empirical results indicate that economic growth and openness reduce deforestation and improve environmental quality. This is confirmed by the work of Hua and Boateng.^13^ Based on a sample of 167 countries observed over the period 1970–2007, the authors reported a strong association between economic growth, trade and environment quality. Their results support the environmental Kuznets curve (EKC). The EKC was first proposed by Simon Kuznets in the 1950s and '60s to graphically study the relationship between economic development and inequality. It argues that inequality increases at the initial stage of economic growth and then gradually declines when the economy reaches a certain level of per capita income. The same EKC hypothesis was confirmed in the environment growth relationship. It represents the relationship between environmental degradation and per capita gross domestic product (GDP). The EKC exists when the state of the environment worsens at the initial stage of economic growth and then gradually improves as the economy reaches a certain level of per capita income.^19^

Samimi *et al.* studied the relationship between environmental performance and economic growth in the Organization of Islamic countries between 2006–2008.^20^ Although that the level environmental performance index (EPI) is not satisfactory in these countries, the authors reported a positive association between EPI and growth. The positive relationship is more pronounced in countries with level economic growth. In order to analyze the linkage between environmental performance and income level in the world economy in 2016, Neagu *et al.* used a sample of 166.^21^ They found a positive association between per capita GDP and the EPI.

**Abbreviations**
*DCPS*: Domestic credit to the private sector
*EKC*: Environmental Kuznets curve
*EPI*: Environmental performance index
*FDI*: Foreign direct investment
*GDP*: Gross domestic product
*MEAS*: Middle East countries
*MENA*: Middle East and North Africa
*NAF*: North Africa countries
*PSTR*: Panel smooth transition regression

In contrast to findings supporting a positive linkage between environmental performance and economic growth, several studies have supported the opposite view. For example, Gumilang *et al.* used a database related to 57 sectors and 87 regions in Indonesia in 2002 to explore the impact of growth on the environment.^17^ They argued that rapid growth generally leads to a deterioration of the environment. Al-Mulali *et al.* found a similar result in a European context.^12^ The authors used a sample of 23 European countries from 1990–2013. They found that GDP growth and financial development increase carbon dioxide emission and deteriorate environmental quality. Therefore, trade openness reduces carbon dioxide emissions. Similarly, Feridun *et al.* found that real GDP and trade liberalization were positively associated with pollution.^16^

More recently, in order to study the economic growth-pollution relationship, Kong and Khan used a sample of 29 countries divided into two sub-samples (14 developed and 15 developing countries) from 1977–2014.^9^ They used two empirical approaches. The first one is based on generalized method of moments regressions and the second one is conducted using panel cointegration and the vector error correction model. Results of generalized method of moments analysis confirm the EKC hypothesis. Moreover, in the finding of the vector error correction model, the short-run analysis shows a unidirectional causality from per capita GDP growth to manufacturing industries and coal rent.

In order to test the presence of the EKC hypothesis, Saud *et al.* used a sample of 59 Belt and Road Initiative countries from 1980–2016 to explore the effect of financial development, economic growth, electricity consumption, and trade openness on environmental quality.^15^ The results of the dynamic seemingly unrelated regression method showed that financial development, foreign direct investment, and trade openness improves environmental quality. However, an increase in economic growth and electricity consumption deteriorates environmental quality.

Based on a sample of Gulf Cooperation Council countries during 1980–2012, Bader and Ganguli aimed to check the validity of the EKC between per capita GDP and indicators of environmental quality.^14^ They performed a panel cointegration analysis and Granger causality test to assess this relationship. The findings indicated the absence of an EKC curve for most of the Gulf Cooperation Council. However, the results supported the existence of a U-shaped relationship for some kingdoms like Bahrain and Saudi Arabia.

Empirical results indicate a threshold effect in the environmental performance and economic growth relationship. More specifically, the impact of environmental performance and economic growth is positive and significant only if a certain threshold level has been attained. Until then, the effect remains negative.

To the best of our knowledge, no previous study has examined the non-linear relationship between environmental performance and economic growth. This study contributes to the existing literature in several ways. First, contrary to previous studies exploring the linear association between environmental performance and economic growth, the current study is a non-linear analysis that defines the optimal threshold of EPI that can affect economic growth in Middle Eastern and North African (MENA) countries.^8–10^,^22^ Second, the whole sample of the MENA region is divided into two sub-samples: Middle Eastern (MEAS) countries and North African (NAF) countries. Contrary to earlier studies related to MENA region that tested one block of countries, the present study takes into account several economic, social and environmental differences.

## Methods

To investigate the non-linear relationship between environmental performance and economic growth, we used a sample of 14 MENA countries over the period of 2002–2018. Countries in the MENA region differ with regard to the import or export of oil. They include countries characterized by high-income as well as middle- and low-income, and range from high levels of growth to low and/or negative economic growth rates. Taking into account the heterogeneity of some countries in the MENA region and to increase the value of the results comparison, the whole sample was divided into two sub-samples. The first sub-sample consists of MEAS countries and covers 9 countries (Bahrain, Iraq, Jordan, Kuwait, Lebanon, Oman, Qatar, Saudi Arabia and United Arab Emirates) and the second sub-sample are the 5 countries that comprise the North Africa countries (Algeria, Egypt, Libya, Morocco and Tunisia) using the panel smooth transition regression (PSTR) model as an econometric approach.^23^,^24^

In the present study, environmental performance as a dependent variable was measured by the EPI, and economic growth is proxied by the growth rate of per capita GDP. As classical and key determinants of growth, the model used in the present study includes four independent variables: domestic credit to the private sector (DCPS), gross fixed capital formation in % GDP as a proxy of domestic investment, foreign direct investment (FDI) net inflow, and trade openness. All of the variables used in this study were retrieved from the World Development Indicators except for the EPI, which was collected from the Yale Center for Environmental Law and Policy.^25^

### Panel smooth transition regression approach

Contrary to previous studies exploring the linkage between environmental performance and economic growth using a linear model,^8–10^ the current study used a non-linear model based on the PSTR model proposed by Hua *et al*.^13^ This model assumes that the relationship between EPI and economic growth may be non-linear, exhibiting a threshold effect in the EPI-growth relationship. The PSTR model can be written by Equation 1.

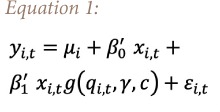
where, *i= 1, . . . , N*, and *t= 1, . . . , T*, with *N* and *T* denoting the cross-section and time dimensions of the panel, respectively. *y*_*i,t*_ is the dependent variable. *u*_*i*_ indicates the vector of the individual fixed effects and *g(q_i,t_,γ,c)g* is the function of transition which depends on the transition variable of transition (*q*_*it*_), to the parameter of threshold (*C*) and to the smooth transition parameter *(γ)*. *x*_*i,t*_ *= (x*^*1 *^_*i,t*_ *,.........,x*^*k*^_*i,t*_) is a vector of *k* explanatory variables and *ϵ*_*i,t*_ is a random disturbance. *β*_*0*_ and *β*_*1*_ indicate the parameter vector of the linear model and the non-linear model, respectively. One of the initial hypotheses of the PSTR model that must be confirmed is non-linearity between the dependent and the transition variable. Secondly, the number of regimes was determined. In other words, the model in the present study may have one threshold and two regimes or two thresholds and three regimes, etc. The third step involves determining the optimal threshold. Finally, we estimate the PSTR model that defines the effect of the transition variable on the dependent variables within two intervals; below and above the threshold.

To investigate the link between environmental performance and economic growth in the MENA region, the following PSTR model is presented in Equation 2.

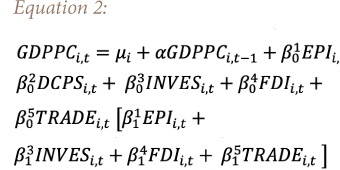



In Equation 2, the transition variable is the EPI and the dependent variable is the growth rate of GDP per capita, where DCPS is domestic credit to the private sector, INVES is gross fixed capital formation in % GDP as a proxy of domestic investment, FDI is foreign direct investment net inflow and TRADE is trade openness. Variable definitions are displayed in Table 1.

**Table 1:** Variable Definition

**Variable**	**Definition**	**Source**
GDPPC	GDP per capita growth (annual %)	WDI (2002–2018)^26^
EPI	Environmental performance index	Yale Center for Environmental Law and Policy^25^
DCPS	Domestic credit to private sector (% of GDP)	WDI (2002–2018)^26^
INVES	Gross fixed capital formation (% of GDP)	WDI (2002–2018)^26^
FDI	Foreign direct investment, net inflows (% of GDP)	WDI (2002–2018)^26^
TRADE	Trade openness (% GDP)	WDI (2002–2018)^26^

Abbreviation: WDI, World Development Indicators.

## Results

Table 2 presents data from the descriptive analysis. Each variable is presented with its average, maximum and minimum value. Furthermore, the whole sample was analyzed according to the two sub-samples: MEAS countries and NAF countries.

**Table 2:** Descriptive Statistics

**MEAS countries**						**NAF countries**				
	
**Variables**	**%**	**Mean Increase (%)**	**Standard deviation**	**Min**	**Max**	**%**	**Mean Increase (%)**	**Standard deviation**	**Min**	**Max**
GDPPC	145	0.225	6.796	−34.898	50.122	80	2.679	16.212	−62.225	122.968
EPI	147	55.749	10.393	32.610	86.000	69	54.321	8.357	41.320	77.280
DCPS	140	54.746	25.565	1.267	105.475	80	41.741	27.195	6.201	95.507
INVES	145	22.115	5.365	5.361	34.523	80	25.140	7.408	8.949	43.049
FDI	143	3.963	4.252	−3.152	23.537	78	2.451	1.964	−0.324	9.424
TRADE	145	106.407	30.256	51.043	191.878	80	77.531	22.770	30.247	138.898

Abbreviation are defined in Table 1

As shown in Table 2, on average, MEAS countries recorded a growth rate of 0.225% per capita GDP compared to 2.679% for NAF countries. With regard to the EPI, the results indicate no strong difference in the mean value of this index for the two groups of countries from 2002–2018. The MEAS countries in the present study had an average EPI value of 55.749 compared to 54.321 for the NAF countries. However, the MEAS countries showed a high level of environmental performance (86) compared to the NAF countries (77.280). In addition, the average value of DCPS was 54.746% for the MEAS countries and 41.741% for the NAF countries in the present study.

The levels of domestic and foreign investment showed differences between the two groups of countries. The average value of domestic investment (gross fixed capital formation (% of GDP)) was 22.115% and 25.140%, respectively, for the MEAS and the NAF countries. However, MEAS countries attract more FDI (3.963%) than NAF countries (2.451%). Trade openness showed a mean value of 106.407% in the MEAS countries compared to 77.531% in the NAF countries.

### Correlation matrix

Table 3 presents the results of the analysis of the absence of multicollinearity between the independent variables used in this study.

**Table 3:** Correlation Matrix

	**GDPPC**	**EPI**	**DCPS**	**INVES**	**FDI**	**TRADE**
GDPPC	1.0000					
EPI	0.0214	1.0000				
DCPS	−0.1552	−0.0041	1.0000			
INVES	0.0152	0.0506	0.0646	1.0000		
FDI	0.0206	−0.0778	0.3307	0.1631	1.0000	
TRADE	−0.0691	−0.0010	0.3189	0.0682	0.2658	1.0000

Abbreviations: GDPPC, GDP per capita growth; INVES, gross fixed capital formation (% of GDP); TRADE, trade openness (% GDP).

Table 3 shows a weak level of correlation between the independent variables introduced in our econometric model, confirming the absence of multicollinearity.

### Pre-test results

Before testing the PSTR model, a test for non-linearity was conducted. Secondly, the number of regimes was checked. The third step consists of determining the optimal threshold.

### Test of linearity

The main objective of this test is to check and confirm that the relationship between environmental performance and economic growth is non-linear. To this end, a test of linearity was conducted against the PSTR model. The null hypothesis is H_0_: and the alternative is H_1_:H. Three statistics were used to confirm whether this relationship is non-linear: the Lagrange Multiplier Wald test, the Lagrange Multiplier F-test and the likelihood ratio test.

Table 4 shows that the null hypothesis is rejected at the 1% and 5% levels for the three tests. Linearity is rejected for the whole sample and the two sub-samples. Statistics of these tests imply that a non-linear relationship exists between environmental performance and economic growth for the whole sample.

**Table 4:** Linnearity Test

**Tests**	**Whole sample**	**MEAS countries**	**NAF countries**
Lagrange Multiplier Wald test	48.101 (0.000)	11.904 (0.0481)	23.259 (0.000)
Lagrange Multiplier F-test	11.541 (0.000)	3.915 (0.0367)	7.801 (0.000)
Likelihood-ratio test	55.289 (0.000)	12.282 (0.0476)	28.790 (0.000)

Values figure in parentheses are the P- values associated to Wald test, F-test and likelihood-ratio test.

### Test of regime number

This test determines the number of transition functions. It aims to check the null hypothesis when the PSTR model has one transition function (*m*=1) against the alternative hypothesis when the model has at least two transition functions (*m*=2). Decisions of this test are based on the likelihood-ratio test and F-test statistics.

Table 5 above indicates that the coefficients are statistically significant at level of 5%, therefore we reject the null hypothesis and conclude that there are at least two transition functions and one threshold for the model.

**Table 5:** Test for the Number of Regimes

**GDPG (transition variable)**	**Whole sample**			**MEAS countries**		**NAF countries**	

**Hypotheses**	**Tests**	**Statistics**	**P-value**	**Statistics**	**P-value**	**Statistics**	**P-value**
*(1)H_0_ : r = 0;H_1_ : r = 0*	LR	21.206	(0.017)	29.356	(0.000)	33.851	(0.000)
	F	2.468	(0.008)	4.978	(0.000)	8.422	(0.000)
*(2)H_0_ : r = 1;H_1_ : r = 2*	LR	48.245	(0.000)	52.012	(0.000)	62.058	(0.000)
	F	61.784	(0.000)	69.612	(0.000)	77.793	(0.000)

Abbreviations: LR, likelihood-ratio test; F, F-test.

### Threshold values

After checking the non-linearity hypothesis environmental performance and economic and identifying the number of regimes, the third step consists of searching the threshold of EPI that can affect the level of per capita GDP in the whole sample and the two sub-samples. Table 6 presents the optimal thresholds.

**Table 6:** Results of Threshold Values

**Tests**	**Whole sample**	**MEAS countries**	**NAF countries**
γ	2.5000	5.0000	5.0000
***C***	**61.710**	**46.669**	**48.528**
AIC	4.178	3.284	3.511
BIC	4.380	0.3.543	3.846

Abbreviations: ***C***, parameter of threshold; γ, smooth transition parameter, AIC, Akaike information criterion; BIC, Bayesian information criterion.

Table 6 indicates that the optimal thresholds of EPI differ between the two groups of countries. For example, the threshold is 61.710 for the whole sample, 46.669 for the MEAS countries and 48.528 for the NAF countries. A comparison of these thresholds suggests that in order to act positively on the level of growth, NAF countries require greater environmental performance compared to MEAS countries.

Table 7 presents the estimation of the PSTR model for the whole sample of 14 countries and the two sub-samples of the MEAS and NAF countries from 2002–2018.

**Table 7:** Estimated Results of the PSTR Model

	**Whole sample**			**MEAS countries**			**NAF countries**		

**Variable**	**Coefficient**	**T-Statistic**	**Significance**	**Coefficient**	**T-Statistic**	**Significance**	**Coefficient**	**T-Statistic**	**Significance**
**First regime**	**EPI < 61.710**			**EPI < 46.669**			**EPI < 48.528**		

EPI	0.062	0.294	0.769	−0.747	−2.134	0.035^**^	−2.835	−9.294	0.000^***^
DCPS	−0.084	−1.392	0.166	−0.055	−0.293	0.770	1.371	13.116	0.000^***^
INVES	−0.122	−0.837	0.404	0.375	1.740	0.084	0.237	0.843	0.403
FDI	0.076	0.315	0.753	0.865	0.314	0.754	2.938	2.666	0.010^***^
TRADE	−0.085	−1.661	0.098^*^	−0.489	−4.924	0.000^***^	0.148	1.121	0.214
**Second regime**	**EPI > 61.710**			**EPI > 46.669**			**EPI > 48.528**		

EPI	0.451	3.185	0.002^***^	0.631	1.830	0.070^*^	2.910	11.731	0.000^***^
DCPS	−0.475	−6.415	0.000^***^	−0.038	−0.203	0.840	−1.470	−15.199	0.000^***^
INVES	1.904	6.048	0.000^***^	0.404	1.442	0.152	0.278	0.769	0.445
FDI	1.063	2.244	0.026^**^	0.785	0.284	0.777	2.460	2.116	0.039^**^
TRADE	0.203	3.633	0.000^***^	0.404	3.833	0.000^***^	0.047	0.745	0.432
AIC	4.178			3.284			3.511		
BIC	4.380			3.543			3.846		
R^2^	0.3688			0.3184			0.8811		
DW	2.058			1.799			1.963		
**Obs**	**238**			**153**			**85**		

^***^, ^**^ and ^*^indicated level of significance respectively at 1%, 5% and 10%.

Abbreviations: T-Stat., t-statistic; INVES, gross fixed capital formation (% of GDP); TRADE, trade openness (% GDP); AIC, Akaike information criterion; BIC, Bayesian information criterion; Obs, number of observation; R^2^, R squared, DW, Durbin Watson Test for autocorrelation.

Subsequently, Table 7 indicates the optimal threshold; EPI exerts a negative and significant effect on per capita GDP. This negative effect was confirmed for the whole sample as well as for the MEAS countries and the NAF countries separately. However, surpassing the threshold of 61.710 for the whole sample and 46.669 for the MEAS countries and 48.528 for the NAF countries, the effect of the EPI on the level of growth becomes positive and significant. This indicates that an improvement in environmental performance significantly increases the level of growth in the MENA region.

## Discussion

Below the optimal threshold, domestic credit to the private sector was found to be positively and significantly correlated with the dependent variable for the NAF countries only. However, there was no significant effect for the other group of countries. Domestic credit to the private sector has been widely used as a proxy for financial development. Several empirical studies have supported the hypothesis of finance-led growth. Making credit conditions less constraining and increasing access to finance may work to stimulate investment. In addition, access to credit enhances the productive capacity of businesses. Businesses and enterprises with adequate financial access have greater potential for growth. In this case, credits granted to the private sector would grow output. These results corroborate the findings of Hamdi *et al*., Christopoulos and Tsionas, Loayza and Ranciere that support the positive effect of domestic credit to private sector on the economic growth.^24^,^27^,^28^ For the second regime, when surpassing the optimal threshold of EPI of 48.528, the effect of DCPS on the dependent variable becomes negative and significant.

Within the first regime, domestic investments as proxied by the gross fixed capital formation, were not found to exert any significant effect on the level of growth for the whole sample and the two sub-samples. However, above the optimal threshold, domestic investment exerts a positive and significant effect on GDP per capita for the whole sample only. For the two sub-samples, the effect is still not significant. On the contrary, FDI exerts a positive effect for the NAF countries only either below or above the EPI threshold of 48.528. In these countries, FDI is considered a driver for economic growth. It is also considered to be an important mechanism resulting in a technology transfer that improves the competitiveness of local employees and firms. Moreover, FDI can create more job opportunities which offer more satisfactory wages that are able to spur per capita GDP and improve living standards. These findings are in line with numerous studies.^29–32^

The effect of trade openness on per capita GDP differs from the first regime to the second regime. In other words, below the optimal threshold of 61.710 for the whole sample and 46.669 for the MEAS countries, the effect of this variable is negative and significative. Without reaching the optimal threshold of EPI, trade is considered one of the biggest contributors to pollution, threatening human health and decreasing the level of growth. Below these thresholds, trade openness significantly decreases the level of per capita growth. However, surpassing these thresholds, openness seems to exert a positive and significant effect on growth levels. The environmental quality is preserved in countries that reach the EPI threshold, where trade openness can boost economic growth.

## Conclusions

Motivated by the assumption that the relationship between environmental performance and economic growth might be non-linear, we used a sample of 14 countries located in the MENA region to study this association. The whole sample was divided into two sub-samples in order to take into consideration the heterogeneity between countries in the same region. The PSTR method was performed to test the non-linear relationship.

Empirical results indicate a threshold effect in the environmental performance and economic growth relationship. Furthermore, the threshold value differed from one group of countries to another. For example, the optimal EPI thresholds are 61.710 for the whole sample, 46.669 for the MEAS countries and 48.528 for the NAF countries. More specifically, we found that the impact of environmental performance and economic growth in the three groups is positive and significant only if the optimal threshold has been attained. Until that point, the effect remains negative.

These results are of great importance to policymakers since they determine the optimal EPI level required to act positively on the level of economic growth. In the present study, MEAS countries had an average EPI value of 55.749 and 54.321 for the NAF countries, indicating that on average, the two groups of countries have reached an optimal EPI level. The MEAS and NAF countries should seek to improve their EPI, as economic growth is improved above the optimal threshold. Trade openness in the MEAS countries should be more dynamic and governments should work to support this activity since trade openness above the optimal threshold of EPI exerts a positive impact on economic growth. The NAF countries should work to attract more foreign direct investment in order to grow output. This is possible with improvement of the business environment along with institutional and infrastructure quality. The NAF countries should also seek to manage and control domestic credit to the private sector since this significantly increases the level of growth.
